# Supplementary material for: A stochastic multicellular model identifies biological watermarks from disorders in self-organized patterns of phyllotaxis
Source: eLife. 2016 Jul 6;5:e14093. doi: 10.7554/eLife.14093 (PMC4947393; doi:10.7554/eLife.14093)
Supplement: Figure 5—source data 1. — This file contains a table showing the variation of permutation intensities with model parameters. We ran simulations using the SMPmacro-max model for different parameter values where the local minima of inhibition profile indicate the potential initiation sites (for this, the inhibitory field values were estimated at 360 sampling points regularly distributed around the periphery of the central zone). We then used the combinatorial model (Refahi et al., 2011) to detect permutations in the simulated sequences. The model has mainly three parameters, β, E∗ and Γ. For each parameter value of β, E∗ and Γ, we run sixty simulations. Each simulation generated a sequence of 25 divergence angles. We then analyzed the sequences using the combinatorial model to detect permutation patterns. DOI: http://dx.doi.org/10.7554/eLife.14093.012 [file elife-14093-fig5-data1.pdf]

$\Gamma \mid E^*$	β	9	10	11	12	13	14
0.475	1	138.3<br>51.8(29.9,21.8)	137.8<br>44.5(27.3,17.2)	137.6<br>38.8(23.8,15.0)	137.4<br>37.8(26.4,11.5)	137.8<br>34.8(24.4,10.4)	137.4<br>26.3(21.9,4.4)
	1.2	137.8<br>42.3(27.0,15.3)	137.8<br>34.8(24.4,10.4)	137.7<br>33.5(23.0,10.5)	137.6<br>27.1(18.3,8.8)	137.6<br>23.6(18.0,5.6)	
	1.4	137.8<br>36.8(23.4,13.4)	137.9<br>34.4(23.6,10.9)	137.9<br>30.4(21.2,9.2)			
0.525	1	137.4<br>46.9(26.9,20.1)	137.5<br>39.4(24.6,14.8)	137.3<br>37.2(24.5,12.7)	137.4<br>32.4(24.6,7.8)	137.4<br>28.5(22.7,5.8)	137.4<br>24.0(19.1,4.9)
	1.2	137.5<br>39.7(23.1,16.6)	137.8<br>33.6(24.3,9.3)	137.6<br>28.7(19.9,8.8)	137.4<br>26.5(21.6,4.9)	137.5<br>18.7(15.7,3.0)	
	1.4	138.0<br>33.3(22.3,11.0)	137.8<br>28.4(20.6,7.9)	137.9<br>27.5(18.7,8.8)			
0.575	1	137.0<br>46.4(27.0,19.4)	137.5<br>38.8(27.9,10.9)	137.2<br>28.5(21.6,6.9)	137.3<br>28.1(23.3,4.8)	137.1<br>21.0(19.4,1.5)	137.5<br>24.8(23.0,1.8)
	1.2	137.5<br>31.4(24.4,7.0)	137.6<br>30.8(25.3,5.5)	137.3<br>24.2(21.7,2.5)	137.5<br>21.0(19.7,1.2)	137.3<br>18.1(16.3,1.8)	
	1.4	137.6<br>31.1(24.9,6.2)	137.7<br>25.3(19.0,6.3)	137.5<br>19.4(18.9,0.5)			
0.625	1	135.9<br>42.4(22.9,19.5)	136.2<br>35.4(25.5,9.8)	136.8<br>27.7(22.8,4.9)	136.8<br>24.0(20.5,3.4)	136.8<br>20.9(18.4,2.5)	136.9<br>18.6(17.2,1.5)
	1.2	136.8<br>28.3(21.2,7.0)	136.7<br>22.1(17.6,4.5)	136.8<br>21.5(18.6,2.9)	136.8<br>14.8(14.4,0.4)	136.9<br>10.9(10.7,0.2)	
	1.4	136.9<br>21.1(19.5,1.6)	137.0<br>16.1(14.2,1.8)	136.8<br>13.1(12.1,1.1)			
0.65	1	136.0<br>36.9(24.1,12.9)	136.4<br>30.0(22.3,7.7)	136.6<br>23.9(19.2,4.7)	136.8<br>18.4(15.7,2.8)	136.8<br>15.3(13.9,1.4)	136.96<br>11.4(11.0,0.4)
	1.2	136.7<br>27.5(23.6,3.9)	136.8<br>21.6(19.7,1.9)	136.8<br>14.6(13.8,0.7)	136.8<br>10.6(9.7,0.8)	136.9<br>7.5(7.3,0.2)	
	1.4	136.9,0.7<br>18.6(16.5,2.0)	136.9,0.5<br>14.9(13.9,1.0)	136.8,0.3<br>8.5(7.7,0.7)			
0.7	1	135.5<br>35.9(24.6,11.3)	136.6<br>29.8(23.1,6.8)	136.4<br>26.4(22.2,4.2)	136.8<br>20.6(18.1,2.5)	137.1<br>14.3(12.3,1.9)	137.1<br>12.3(11.6,0.7)
	1.2	136.7<br>26.5(20.2,6.3)	136.7<br>21.5(18.3,3.2)	137.0<br>12.5(11.2,1.3)	137.2<br>12.7(11.9,0.7)	137.0<br>9.3(8.6,0.7)	
	1.4	137.0<br>16.6(16.0,0.6)	137.0<br>13.1(11.8,1.2)	137.0<br>10.1(9.7,0.4)			

Table 1: Average angle (top value) and permutation frequency values (bottom values), for $0.475 \leq \Gamma \leq 0.7$)

$\Gamma \mid E^*$	β	9	10	11	12	13	14
0.75	1	136.5<br>32.6(21.6,11.0)	136.2<br>30.7(21.2,9.5)	136.7<br>22.8(18.1,4.7)	137.3<br>15.5(11.9,3.5)	137.6<br>11.4(10.5,0.9)	137.4<br>10.6(10.6,0.0)
	1.2	136.9<br>22.4(19.6,2.8)	136.5<br>21.4(17.4,4.0)	136.6<br>11.1(10.1,1.0)	137.3<br>10.0(9.2,0.9)	137.3<br>6.9(6.9,0.0)	
	1.4	136.5<br>16.5(14.9,1.7)	136.9<br>14.8(12.4,2.4)	137.0<br>8.7(8.3,0.4)			
0.8	1	137.2<br>32.3(23.2,9.1)	136.6<br>29.0(21.2,7.8)	137.3<br>19.8(14.1,5.7)	137.7<br>14.7(12.4,2.3)	138.3<br>10.7(9.7,0.9)	137.9<br>10.5(9.6,0.9)
	1.2	136.4<br>25.6(19.7,5.9)	136.6<br>21.5(18.1,3.4)	137.4<br>11.7(10.4,1.2)	136.8<br>13.6(12.4,1.1)	137.7<br>7.2(7.0,0.2)	
	1.4	136.5<br>15.9(13.5,2.4)	137.2<br>11.2(10.8,0.4)	137.2<br>8.3(8.3,0.0)			
0.85	1	138.5<br>22.7(15.7,7.0)	138.5<br>17.6(13.5,4.1)	138.9<br>10.9(10.5,0.4)	138.8<br>9.4(9.2,0.2)	138.7<br>8.2(7.8,0.4)	139.1<br>4.0(3.6,0.4)
	1.2	137.7<br>18.1(14.5,3.6)	138.0<br>11.9(11.0,0.9)	138.3<br>9.2(8.8,0.4)	138.2<br>7.3(6.9,0.4)	138.5<br>4.0(4.0,0.0)	
	1.4	137.6<br>12.2(11.5,0.7)	137.6<br>9.6(8.2,1.4)	137.9<br>4.5(4.5,0.0)			
0.9	1	138.7<br>20.6(14.5,6.1)	139.3<br>15.8(10.7,5.1)	139.5<br>10.7(9.0,1.6)	139.5<br>7.2(6.1,1.1)	139.7<br>5.5(5.3,0.2)	140.0<br>2.3(2.3,0.0)
	1.2	138.3<br>17.8(14.8,3.1)	138.6<br>11.4(8.3,3.1)	138.9<br>8.9(8.1,0.8)	138.7<br>7.1(6.9,0.2)	139.1<br>4.0(4.0,0.0)	
	1.4	138.1<br>11.9(11.2,0.7)	138.4<br>8.3(8.1,0.2)	138.5<br>4.5(4.5,0.0)			
0.95	1	139.8<br>17.7(14.3,3.3)	139.9<br>11.7(9.8,1.8)	139.7<br>8.9(7.6,1.2)	140.1<br>4.8(4.4,0.4)	139.9<br>4.4(4.2,0.2)	139.9<br>2.7(2.7,0.0)
	1.2	139.4<br>10.3(9.3,1.0)	139.5<br>6.0(6.0,0.0)	139.3<br>5.3(5.3,0.0)	139.6<br>4.1(3.8,0.4)		
	1.4	139.0<br>8.2(7.5,0.7)	138.9<br>6.5(6.5,0.0)	139.3<br>2.9(2.9,0.0)			
0.975	1	140.2<br>17.7(16.3,1.4)	139.5<br>7.7(6.9,0.8)	140.3<br>7.3(7.3,0.0)	140.2<br>3.7(3.7,0.0)	140.3<br>3.5(3.5,0.0)	140.3<br>1.2(1.2,0)
	1.2	139.7<br>10.0(8.6,1.4)	139.9<br>5.7(5.1,0.6)	139.8<br>4.0(3.6,0.4)	140.1<br>1.7(1.7,0.0)	140.1<br>0.8(0.8,0.0)	
	1.4	139.3<br>8.0(7.9,0.2)	139.5<br>4.8(4.6,0.2)	139.5<br>2.6(2.6,0.0)			

Table 2: Average angle (top value) and permutation frequency values (bottom values), for $0.75 \leq \Gamma \leq 0.975$
